# Biomarker-based diagnosis of ventilator-associated pneumonia using serum and bronchoalveolar lavage fluid levels of presepsin, procalcitonin, and lipopolysaccharide-binding protein

**DOI:** 10.3389/fcimb.2026.1747971

**Published:** 2026-02-25

**Authors:** Fang-Hui Ni

**Affiliations:** Emergency Department, Pingyang Hospital Affiliated to Wenzhou Medical University, Wenzhou, China

**Keywords:** diagnosis, lipopolysaccharide-binding protein, mechanical ventilation, presepsin, procalcitonin, ventilator-associated pneumonia

## Abstract

**Objective:**

Mechanically ventilated patients are often confronted with ventilator-associated pneumonia (VAP), showing an increased risk of mortality. Early identification of biomarkers associated with VAP may ease the diagnosis and guide preventive interventions. In this study, we investigated the diagnostic value of VAP using serum and bronchoalveolar lavage fluid (BALF) levels of presepsin, procalcitonin (PCT), and lipopolysaccharide-binding protein (LBP).

**Methods:**

The serum and BALF samples were collected from 300 consecutive mechanically ventilated patients with a clinical suspicion of VAP by bronchoscopy. Among these 300 patients, 126 patients had confirmed VAP, while 174 did not meet the criteria for VAP.

**Results:**

The reference ranges of serum presepsin, PCT, and LBP in patients with confirmed VAP were all higher than those in patients with VAP criteria not fulfilled (*p* < 0.0001). The reference ranges of BALF presepsin and LBP were both higher in patients with confirmed VAP than those in patients with VAP criteria not fulfilled (*p* < 0.0001), whereas the reference range of BALF PCT did not differ between the two groups (*p* = 0.202). Subgroup analysis based on main pathologies found higher levels of serum LBP, BALF presepsin, and LBP in the gram-negative group than in the gram-positive group (*p* < 0.001). Serum levels of presepsin, PCT, and LBP for VAP diagnosis presented AUC values of 0.81, 0.76, and 0.78, respectively. Combined analysis of serum presepsin and LBP for the diagnostic evaluation of VAP showed an AUC of 0.88, and combined analysis of the three serum levels for the diagnostic evaluation of VAP showed an AUC of 0.90. The BALF levels of presepsin and LBP for VAP diagnosis presented AUC values of 0.85 and 0.84, respectively. Combined analysis of the two BALF levels for the diagnostic evaluation of VAP showed an AUC of 0.92. Combining BALF levels of presepsin and LBP may yield better diagnostic value for the development of VAP in mechanically ventilated patients than serum.

**Conclusion:**

Our findings point to the importance of selecting the correct biological fluid when analyzing molecular diagnostics for definitive VAP among mechanically ventilated patients with suspicion of VAP.

## Introduction

Ventilator-associated pneumonia (VAP) is a significant clinical entity that affects up to 20% of patients requiring mechanical ventilation, leading to an increase in significant morbidity and mortality in the intensive care unit (ICU) (Howroyd et al., 2024). Some risk factors of VAP have been identified, such as older age, tracheostomy, and longer duration of mechanical ventilation (Rosenthal et al., 2025). There is no single best definition for the diagnosis of VAP, and current diagnostic methods rely on combinations of chest radiology, clinical signs and symptoms, and/or quantitative microbiological testing (Fally et al., 2024). Rapid identification of mechanically ventilated patients with an increased risk of VAP may accelerate the diagnosis and develop control measures (Frondelius et al., 2024). Better markers of VAP should accelerate the diagnosis, aid in antibiotic treatment, and evaluate the prognosis.

Biomarker-based diagnosis of VAP has been commonly studied using two sample types: serum and bronchoalveolar lavage fluid (BALF) (Pinilla-Gonzalez et al., 2023; Yang et al., 2025). Procalcitonin (PCT) is the precursor structure of the calcitonin hormone with 116 amino acids, and it has been described as a host-response biomarker with clinical value for diagnosing pneumonia, bacterial peritonitis, and sepsis (Paudel et al., 2020). Previous evidence showed PCT as a pertinent biomarker for VAP diagnosis and a helpful tool for antibiotic discontinuation (Cortes et al., 2021). However, a recent systematic review fails to provide sufficient evidence to support the role of PCT in the routine assessment of patients with VAP (Alessandri et al., 2021). Even in BALF, PCT concentration seemed to be less valuable in discriminating VAP and inflammation (Linssen et al., 2008; Grover et al., 2014). Presepsin, also known as soluble CD14 subtype (sCD14-ST), is a 13-kDa truncated form of soluble CD14 (sCD14) with 64 amino acid residues, which exhibits favorable diagnostic accuracy in sepsis across neonates, children, and adults (Formenti et al., 2024; Peng et al., 2025). Increasing evidence showed a higher diagnostic accuracy offered by presepsin than other conventional biomarkers, including PCT, high-sensitivity C-reactive protein (hs-CRP), and IL-6 during bacterial sepsis (Kweon et al., 2014). The plasma level of presepsin was found to be increased after bacterial infections and decreased following antibiotic treatment in critically ill patients (Galliera et al., 2019). Measurements of presepsin in the samples of tracheal aspirate obtained from intubated newborns suggested that presepsin could serve as a complementary marker in diagnosing early-onset neonatal pneumonia (Savic et al., 2018). Lipopolysaccharide-binding protein (LBP) is an acute-phase protein that plays an essential role in the pulmonary immune response to gram-negative infection (Laryushina et al., 2024). LBP level on the 1st day of suspected sepsis has shown a better diagnostic value of sepsis than PCT and soluble CD14 in critically ill neonates and children (Pavcnik-Arnol et al., 2007). LBP has been studied in combination with PCT and presepsin to diagnose and monitor VAP and sepsis, showing significant clinical value (Mussap et al., 2011; Rumende and Mahdi, 2013). However, there is limited evidence showing combinations of presepsin, PCT, and LBP for VAP diagnosis in mechanically ventilated patients. In this study, we examined serum and BALF levels of presepsin, PCT, and LBP in mechanically ventilated patients with VAP and also compared their diagnostic values alone or in combination.

## Methods

### Population

This prospective observational study included mechanically ventilated patients with a clinical suspicion of VAP in the Intensive Care Unit (ICU) of Pingyang Hospital Affiliated to Wenzhou Medical University between January 2024 and December 2024. The inclusion criteria were duration of mechanical ventilation >48 h and age ≥18 years. The definitive diagnosis of VAP in this study was based on the 2005 American Thoracic Society/Infectious Diseases Society of America guidelines, a Clinical Pulmonary Infection Score (CPIS) ≥6, and positive BALF microbiological findings. The exclusion criteria were VAP caused by viral infections alone, diagnosis of tuberculosis, pulmonary hemorrhage, chronic interstitial pneumonia, acute respiratory distress syndrome, lung tumors prior to mechanical ventilation, the presence of other infections acquired during ICU stay, a high risk for fiberoptic bronchoscopy, thoracic dressings, pregnancy, and refusal to participate. Informed written consent was obtained from the relatives of all ventilated patients. Ethical permission was received from the Ethics Committee of Pingyang Hospital Affiliated to Wenzhou Medical University.

### Sample acquisition and processing

Mechanically ventilated patients suspected of VAP underwent bronchoscopy with bronchoalveolar lavage as part of the routine diagnostic workup. The bronchoalveolar lavage procedures were performed within 12 h when mechanically ventilated patients were suspected of VAP. A fiberoptic bronchoscope (Pentax FB-15H/FB-15X, Pentax Medicals, Tokyo, Japan) was wedged into the segmental or subsegmental bronchus, followed by instillation and recovery of 5–7 aliquots of 20 mL sterile saline 0.9% (0.9% NaCl). The BALF samples were sent to the laboratory within 15 min after bronchoscopy and processed immediately. Microbiological analysis of the BALF samples was performed on blood agar and chocolate agar plates. BALF cytology included total cell counts and differential cell counts. Viruses, including *influenza A*, *influenza A H1*, *influenza A H1-2009*, *influenza A H3*, *influenza B*, *parainfluenza virus* (*types 1*, *2*, *3*, and *4*), *respiratory syncytial virus* (*RSV*) *type A*, *RSV type B*, *human rhinovirus*, *SARS-CoV-2*, *adenovirus*, *coronaviruses* (*229E*, *NL63*, *OC43*, and *HKU1*), *human metapneumovirus*, *Chlamydia pneumoniae*, and *Mycoplasma pneumoniae*, in the BALF or nasopharyngeal swab were tested by the real-time polymerase chain reaction (RT-PCR) using the cobas eplex respiratory pathogen panel 2 kit (Roche Diagnostics, Basel, Switzerland). Blood samples were collected along with bronchoalveolar lavage procedures using EDTA-containing tubes from each ventilated patient prior to bronchoscopy and were immediately centrifuged at 1,500*g* for 30 min to obtain serum.

### VAP definition

The definitive diagnosis of VAP in this study was based on the 2005 American Thoracic Society/Infectious Diseases Society of America guidelines, CPIS ≥6, and positive BALF microbiological findings as follows:

1. 2005 American Thoracic Society/Infectious Diseases Society of America guidelines: Evidence of new or progressive radiological infiltrates after 48 h of endotracheal intubation and at least two of the following signs: body temperature ≥38°C or <36°C, white blood cell (WBC) counts ≥10,000/μL or ≤4,000 cells/mm^3^, and purulent respiratory secretions (American Thoracic, 2005).
2. CPIS ≥6 (Luna et al., 2003): The CPIS includes temperature, blood leukocytes per mm^3^, tracheal secretions, oxygenation PaO_2_/FIO_2_ (mmHg), and chest radiograph. The simplified version of the CPIS is presented in **Table 1**. Higher scores indicate a greater likelihood of VAP.
3. Positive microbiological findings in BALF: A quantitative culture result of BALF ≥10^4^ cfu/mL (Torres and El-Ebiary, 2000).

**Table 1:** The simplified version of the CPIS.

Parameter	Point
Temperature, °C	
36.5–38.4	0
38.5–38.9	1
≥39 or ≤36	2
White blood cell count, ×10^3^/μL	
4.0–11.0	0
>11.0 or <4.0	1
Tracheal secretions	
Few	0
Moderate	1
Large	2
Purulent	+1
Oxygenation PaO_2_/FIO_2_, mmHg	
>240 or presence of ARDS	0
≤240 and absence of ARDS	2
Chest radiograph	
No infiltrate	0
Patchy or diffuse infiltrate	1
Localized infiltrate	2

ARDS, acute respiratory distress syndrome.

### Presepsin, PCT, and LBP determinations

The BALF supernatants were obtained by centrifugation at 3,000*g* for 15 min. Presepsin (pg/mL), PCT (ng/mL), and LBP (μg/mL) in the BALF supernatants and serum were determined by the enzyme-linked immunosorbent assay using the kits for presepsin (ml059920), PCT (ml106700), and LBP (ml105950) (Shanghai Enzyme-Linked Biotechnology, China). The normal reference values of presepsin, PCT, and LBP concentrations in serum were 0–300 pg/mL, 0–0.5 ng/mL, and 0–10 μg/mL, respectively (Morgenthaler et al., 2002; Kopp et al., 2016; Kang et al., 2023).

### Statistics

After evaluation of normality assumptions through the Shapiro–Wilk tests, quantitative data were described as either mean with standard deviation (SD) or median with interquartile range (IQR). Data expressed as mean with SD were analyzed using parametric tests (independent *t*-test), whereas data expressed as median with IQR were analyzed using non-parametric tests (Mann–Whitney *U* test). Categorical data were reported as numbers with percentages (%) and analyzed using the chi-squared test. The receiver operating characteristic (ROC) curves and their summary statistics [area under the curve (AUC)] with 95% confidence intervals (CIs) were used to evaluate the diagnostic values of serum and BALF levels of presepsin, PCT, and LBP for VAP. Using multiple logistic regression, presepsin, PCT, and LBP were combined to yield a diagnostic value. All statistical analyses were conducted using IBM SPSS Statistics 27.0 for Windows (IBM, Armonk, NY, USA), and a significant difference was indicated by a *p*-value of <0.05 (two-tailed).

## Results

### Patient characteristics

In the study period, BALF samples with paired serum samples were collected from 300 mechanically ventilated patients suspected of VAP. Among these 300 patients, 126 (42.0%) had confirmed VAP, including 71 cases (56.3%) caused by gram-negative microorganisms, 52 cases (41.3%) caused by gram-positive microorganisms, and 3 cases (2.4%) caused by fungi. Among the 126 patients with confirmed VAP, there were 17 cases with viral co-infections. The distribution of the identified pathogens in patients with confirmed VAP is presented in **Supplementary Table 1**. Patient characteristics are listed in **Table 2**, stratified by patients with confirmed VAP and those with VAP criteria not fulfilled.

**Table 2:** Patient characteristics, stratified to patients with confirmed VAP and those with VAP criteria not fulfilled.

Characteristic	Patients with confirmed VAP (*n* = 126)	Patients with VAP criteria not fulfilled (*n* = 174)	*p*
Age (years, mean ± SD)	56.3 ± 9.0	57 ± 8.8	0.463
Sex (*n*/%)			0.275
Male	85 (67.5%)	110 (60.9%)	
Female	41 (32.5)	64 (39.1%)	
COPD (*n*/%)	18 (14.3%)	20 (11.5%)	0.487
Immunosuppression (*n*/%)	20 (15.9%)	32 (18.4%)	0.644
Reasons for mechanical ventilation (*n*/%)			0.317
Acute respiratory failure	78 (61.9%)	94 (54.0%)	
AECOPD	4 (3.2%)	13 (7.5%)	
Trauma/postoperative respiratory failure	39 (30.9%)	58 (33.3%)	
Nervous system diseases	5 (4.0%)	9 (5.2%)	
WBC count [×10^9^/L, median (IQR)]	10.5 (5.8–15.3)	9.1 (6.3–13.3)	0.146
hs-CRP [mg/L, median (IQR)]	27.5 (15.6–40.0)	23.5 (11.2–37.3)	0.062
APACHE II score [median (IQR)]	20.0 (13.8–27.0)	18.0 (10.8–27.0)	0.068
IDSA criteria fulfilled (*n*/%)	106 (84.1%)	42 (24.4%)	<0.001
CPIS [median (IQR)]	7.0 (6.0–8.0)	2.0 (1.0–4.0)	<0.001
Duration of mechanical ventilation [days, median (IQR)]	14.0 (9.0–18.0)	12.0 (8.0–17.0)	0.109
Time of sampling [days, median (IQR)]	7.0 (5.0–9.0)	6.0 (4.0–8.0)	0.413
Antibiotic use at the time of sampling	94 (74.6%)	113 (64.9%)	0.078
Mortality (*n*/%)	51 (40.5%)	48 (31.0%)	0.111

Data summarized as median with IQR were analyzed by non-parametric tests (Mann–Whitney *U* test), and those summarized as mean ± SD were analyzed by parametric tests (independent *t*-test). Categorical variables were analyzed using chi-squared tests.

VAP, ventilator-associated pneumonia; AECOPD, acute exacerbation of chronic obstructive pulmonary disease; WBC, white blood cell; CRP, C-reactive protein; APACHE II, Acute Physiology and Chronic Health Evaluation II; CPIS, Clinical Pulmonary Infection Score; SD, standard deviation; IQR, interquartile range.

### Serum levels of presepsin, PCT, and LBP according to VAP

**Figure 1:**
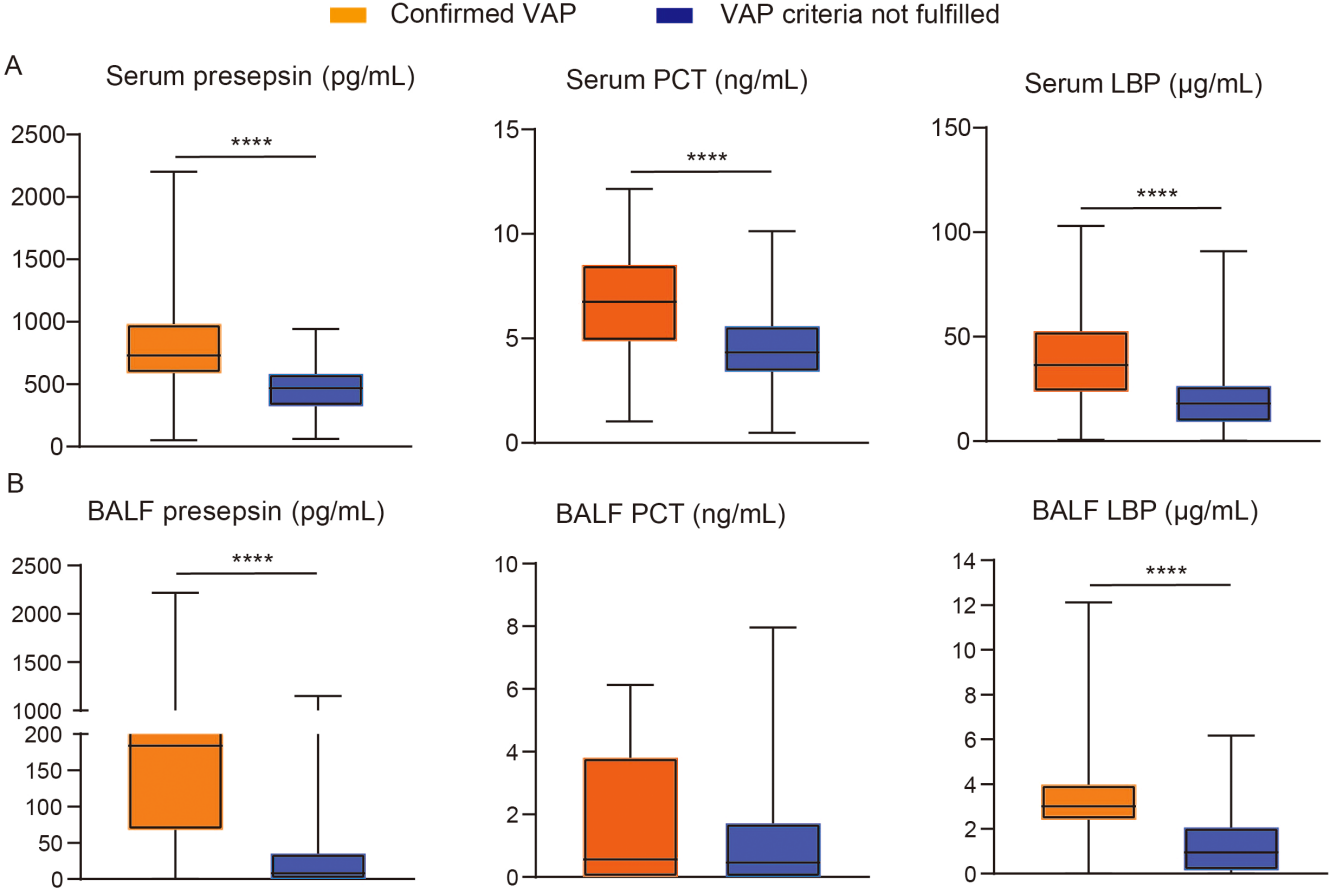
Serum **(A)** and BALF **(B)** levels of presepsin, PCT, and LBP in mechanically ventilated patients with confirmed VAP or VAP criteria not fulfilled. Data summarized as median with IQR were analyzed by non-parametric tests (Mann–Whitney *U* test). ****p < 0.0001.

In patients with confirmed VAP, the median values of serum presepsin, PCT, and LBP were 730.0 pg/mL (IQR: 587.5–982.3 pg/mL), 6.75 ng/mL (IQR: 4.87–8.52 ng/mL), and 36.41 μg/mL (IQR: 23.74–52.67 μg/mL), respectively. In patients with VAP criteria not fulfilled, the median values of the three variables were 468.0 pg/mL (IQR: 323.8–583.3 pg/mL), 4.34 ng/mL (IQR: 3.40–5.59 ng/mL), and 18.00 μg/mL (IQR: 9.29–26.48 μg/mL), respectively. The reference ranges of serum presepsin, PCT, and LBP in patients with confirmed VAP were all higher than those in patients with VAP criteria not fulfilled (*p* < 0.0001; **Figure 1A**). Subgroup analysis of patients with confirmed VAP based on age showed no significant differences in the three variables between subgroups <65 and ≥65 years (the reference ranges are listed in **Table 3**). Subgroup analysis based on main pathologies found higher levels of serum LBP in the gram-negative group than in the gram-positive group (*p* < 0.0001; the reference ranges are listed in **Table 3**), but serum presepsin and PCT were similar.

**Table 3:** The reference ranges of serum and BALF levels of presepsin, PCT, and LBP according to age- and pathology-stratified subgroups in patients with confirmed VAP.

Item	<65 years (*n* = 103)	≥65 years (*n* = 23)	Gram-negative (*n* = 71)	Gram-positive (*n* = 52)
Serum				
Presepsin (pg/mL)	715.0 (556.0–979.0)	791.0 (712.0–1,049.0)	816.0 (602.0–983.0)	703.5 (539.8–943.5)
PCT (ng/mL)	6.76 (4.94–8.32)	6.74 (4.40–9.64)	6.64 (5.03–9.25)	7.03 (4.40–8.08)
LBP (μg/mL)	36.32 (23.72–51.84)	36.65 (27.53–62.56)	40.53 (30.46–60.72)^*^	29.99 (18.75–40.96)^*^
BALF				
Presepsin (pg/mL)	174.0 (55.0–662.0)	253.0 (131.0–728.0)	220.0 (142.0–759.0)^*^	90.5 (17.0–642.3)^*^
PCT (ng/mL)	0.57 (0.00–3.80)	0.56 (0.11–4.13)	0.58 (0.00–4.02)	0.54 (0.00–1.32)
LBP (μg/mL)	2.87 (2.30–3.97)	3.34 (2.46–4.57)	3.34 (2.64–4.53)^*^	2.69 (1.72–3.30)^*^

Data summarized as median with IQR were analyzed by non-parametric tests (Mann–Whitney *U* test).

*p < 0.0001 between the two subgroups.

### BALF levels of presepsin, PCT, and LBP according to VAP

The median values of BALF presepsin, PCT, and LBP were 184.0 pg/mL (IQR: 68.0–678.5 pg/mL), 0.57 ng/mL (IQR: 0.00–3.80 ng/mL), and 3.01 μg/mL (IQR: 2.41–3.97 μg/mL), respectively, in patients with confirmed VAP. The median values of BALF presepsin, PCT, and LBP were 8.0 pg/mL (IQR: 0.0–35.3 pg/mL), 0.47 ng/mL (IQR: 0.0–1.71), and 0.94 μg/mL (IQR: 0.14–2.06 μg/mL), respectively, in patients with VAP criteria not fulfilled. The reference ranges of BALF presepsin and LBP were both higher in patients with confirmed VAP than those in patients with VAP criteria not fulfilled (*p* < 0.0001; **Figure 1B**). However, the reference range of BALF PCT did not differ between the two groups (*p* = 0.202). Age-stratified analysis showed no significant differences in BALF presepsin and LBP between <65 and ≥65 years (the reference ranges are listed in **Table 3**). Pathology-stratified analysis found higher levels of BALF presepsin and LBP in the gram-negative group than in the gram-positive group (*p* = 0.004 and *p* < 0.0001; the reference ranges are listed in **Table 3**), but BALF PCT was similar.

### Diagnosis of the serum levels of presepsin, PCT, and LBP for VAP

**Figure 2:**
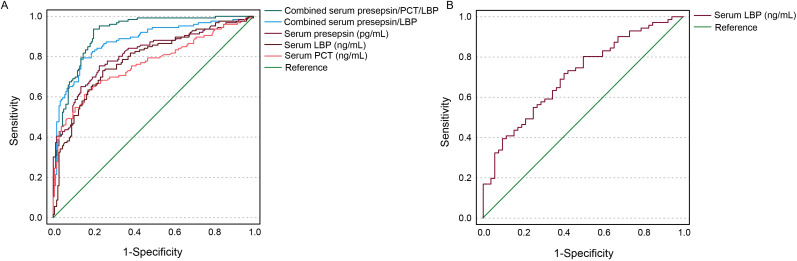
ROC curves showing diagnosis of serum levels of presepsin, PCT, and LBP for VAP **(A)** and gram-negative VAP **(B)**.

The serum levels of presepsin, PCT, and LBP for VAP diagnosis demonstrated AUC values of 0.81 (95% CI: 0.76–0.86; sensitivity: 75.4%; specificity: 73.0%; cutoff value: 590 pg/mL), 0.76 (95% CI: 0.71–0.82; sensitivity: 63.5%; specificity: 82.8%; cutoff value: 6.15 ng/mL), and 0.78 (95% CI: 0.73–0.84; sensitivity: 73.0%; specificity: 75.3%; cutoff value: 26.43 μg/mL), respectively. Combined analysis of the serum presepsin and LBP for the diagnostic evaluation of VAP showed an AUC of 0.88 (95% CI: 0.84–0.92; sensitivity: 78.6%; specificity: 86.2%). Combined analysis of the three serum levels for the diagnostic evaluation of VAP showed an AUC of 0.90 (95% CI: 0.87–0.94; sensitivity: 88.0%; specificity: 80.0%) (**Figure 2A**). The serum level of LBP in diagnosing gram-negative VAP demonstrated an AUC of 0.71 (95% CI: 0.62–0.80; sensitivity: 72.0%; specificity: 60.0%; cutoff value: 33.29 ng/mL) (**Figure 2B**).

### Diagnosis of BALF levels of presepsin and LBP for VAP

**Figure 3:**
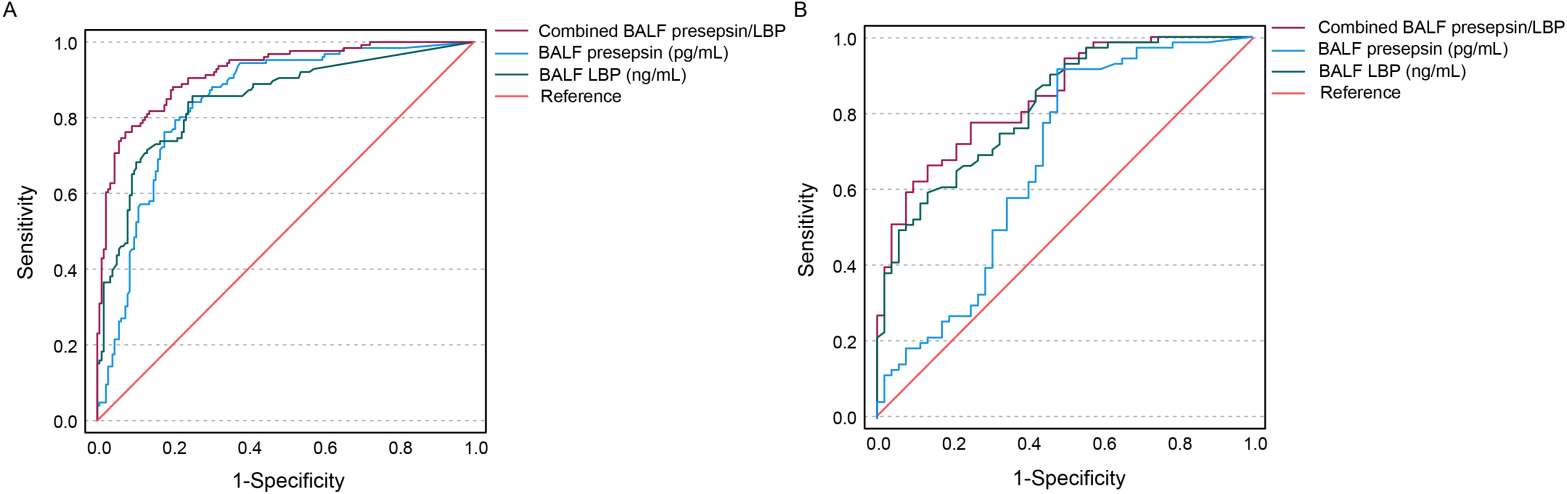
ROC curves showing diagnosis of BALF levels of presepsin, PCT, and LBP for VAP **(A)** and gram-negative VAP **(B)**.

The BALF levels of presepsin and LBP for VAP diagnosis presented AUC values of 0.85 (95% CI: 0.80–0.89; sensitivity: 85.7%; specificity: 74.7%; cutoff value: 31.5 pg/mL) and 0.84 (95% CI: 0.80–0.89; sensitivity: 84.1%; specificity: 74.7%; cutoff value: 2.0 μg/mL), respectively. Combined analysis of the two BALF levels for the diagnostic evaluation of VAP showed an AUC of 0.92 (95% CI: 0.89–0.95; sensitivity: 93.7%; specificity: 80.0%) (**Figure 3A**). The BALF levels of presepsin and LBP in diagnosing gram-negative VAP presented AUC values of 0.67 (95% CI: 0.57–0.77; sensitivity: 88.7%; specificity: 52.0%; cutoff value: 110 pg/mL) and 0.82 (95% CI: 0.75–0.89; sensitivity: 85.9%; specificity: 58.0%; cutoff value: 2.59 μg/mL), respectively. Combined analysis of the BALF presepsin and LBP for the diagnostic evaluation of gram-negative VAP showed an AUC of 0.84 (95% CI: 0.77–0.91; sensitivity: 77.5%; specificity: 75.0%) (**Figure 3B**).

## Discussion

The study demonstrates that combining presepsin and LBP, sampled in BALF, exhibits better diagnostic value for VAP in mechanically ventilated patients than that sampled in serum. Selecting a correct biological fluid for the measurement of specific predictors results in further improvement in a biomarker panel to diagnose the occurrence of VAP in critically ill patients with mechanical ventilation.

The role of PCT as a biomarker of VAP remains controversial in an earlier work. Song et al (Song et al., 2020). demonstrated that serum PCT is an early, sensitive, and highly specific high-risk monitoring index and has an early prediction value for VAP after cardiac valve replacement. However, in the study of Borkowska et al., no association was observed between PCT level and the occurrence of VAP (Zielinska-Borkowska et al., 2012). In our study, we determined a higher baseline level of PCT sampled in serum but not in BALF in patients developing VAP, aligning with a previous study in which serum but not alveolar PCT was helpful for diagnosing VAP and mortality (Duflo et al., 2002). The synthesis of PCT may be associated with an increased production of tumor necrosis factor-α (TNF-α), as both exhibited a similar expression pattern during infection (Mazaheri et al., 2022). In healthy and septic animals, PCT injection did not initiate or enhance the production of TNF-α, while TNF-α injection induced a 25-fold massive and sustained PCT increase (Whang et al., 2000). This suggests that PCT secretion is an “intermediary” rather than a “proximal” event in the sepsis cascade that requires a “primed” inflammatory background to exert its effect (Dahaba and Metzler, 2009). TNF-α concentration was significantly higher systemically than in the BALF of patients with acute respiratory distress syndrome (Agouridakis et al., 2002). Therefore, in our study, systemic cytokine production can explain the increased serum procalcitonin concentrations, whereas the low alveolar procalcitonin release could be explained by the lower production of the local mediators.

The levels of presepsin and LBP were likely to outperform PCT in evaluating infection severity and the prognosis of severe sepsis/septic shock (Mierzchala et al., 2011; Tsujimoto et al., 2018). Presepsin, a cell-surface glycoprotein, is constitutively expressed on the surface of multiple cells, including monocytes, neutrophils, and macrophages (Wu et al., 2019). It is also a high-affinity receptor specific for lipopolysaccharides (LPS) and LBP complexes, regulating LPS-triggered apoptosis (Tsukamoto et al., 2018). When presepsin binds the LBP complex, Toll-like receptor 4 (TLR4)-specific proinflammatory signaling is activated, followed by initiation of host response against infectious agents (Na et al., 2023). Presepsin is produced by proteolytic shedding of the membrane CD14 (mCD14) during cellular activation; thus, presepsin can be detected proportionately in the BALF followed by the release of the LPS–LBP–CD14 complex (Marcos et al., 2010). Although not studied in VAP patients, Mabrey et al (Mabrey et al., 2021). found that higher plasma soluble CD14 subtype was associated with worse clinical outcomes in critically ill patients. LBP, as a component of the bacterial outer membrane, recognizes lipopolysaccharides and transfers to presepsin, which plays a multifunctional role in potentiating the recognition, clearance, and killing of gram-negative bacteria (Taddonio et al., 2015). Its upregulation is considered part of the acute-phase response. Although LBP is known to respond to gram-negative components, a more expanded role for LBP as a general recognition molecule has been shown (Zweigner et al., 2006). Several bacterial surface components, such as lipoteichoic acid, from gram-positive pathogens are also recognized by this molecule. LBP regulated the complexation of lipoteichoic acid with CD14 and its biological activity toward immune cells. Lipoteichoic acid-like glycolipids isolated from spirochetes are recognized by LBP and initiate signaling in the presence of LBP (Schroder and Schumann, 2005). The upregulation of LBP in serum was found in patients with sepsis regardless of gram-negative, gram-positive, or fungal infections, suggesting that LBP may be less specific to gram-negative infection (Blairon et al., 2003). In our study, higher levels of serum LBP were determined in the gram-negative group than in the gram-positive group, suggesting the primary role of LBP in response to gram-negative infections in VAP. However, the diagnostic value of LBP in gram-negative or gram-positive infection was limited in our further subgroup analysis. Rumende et al (Rumende and Mahdi, 2013). demonstrated that combining PCT and LBP could serve as good prognostic markers to predict mortality in patients with VAP. IL-6 alone or in combination with IL-1β led to elevations in LBP and presepsin concentrations in culture supernatants of human bronchial epithelial cells, suggesting that airway epithelial cells produce LBP and presepsin after stimulus treatment (Regueiro et al., 2009). In our study, although presepsin and LBP were increased in both serum and BALF samples, two of them in BALF alone or in combination exhibited better diagnostic value for patients with confirmed VAP compared with their serum counterparts. Furthermore, combined analysis of the BALF presepsin and LBP also conferred a significant diagnostic value for gram-negative VAP.

The study has certain limitations. First, there was a lack of serial sampling to monitor the dynamic status of the studied biomarkers, which may limit their interpretation and translation into clinical applications. Second, the gold standard for diagnosis of pneumonia is still tissue histology; thus, the lack of a VAP gold standard may lead to misclassification of some patients with non-documented bacterial infection. Third, all patients in this study were recruited due to suspicion of VAP, and the absence of a control group of patients without suspected VAP may limit the clinical utility of the biomarkers studied. Fourth, VAP caused solely by viral infections was excluded in this study, which may limit the generalizability of our findings to virus-induced VAP. Fifth, although several indications for mechanical ventilation were reported in the study, the single-center design with a relatively small sample size may still have missed critically ill patients with other causes, which may also limit the generalizability of our results in other settings.

In conclusion, the study demonstrates that presepsin combined with LBP in BALF may be an adjunct to diagnose definitive VAP among mechanically ventilated patients with suspected VAP. Our findings support the incorporation of presepsin and LBP in BALF into the biomarker panel for definitive VAP in critically ill patients.

## Data Availability

The original contributions presented in the study are included in the article/**Supplementary Material**. Further inquiries can be directed to the corresponding author.
