# Supplementary material for: Biomarker-based diagnosis of ventilator-associated pneumonia using serum and bronchoalveolar lavage fluid levels of presepsin, procalcitonin, and lipopolysaccharide-binding protein
Source: Front Cell Infect Microbiol. 2026 Feb 25;16:1747971. doi: 10.3389/fcimb.2026.1747971 (PMC12975765; doi:10.3389/fcimb.2026.1747971)
Supplement: Supplementary file 1 [file Table1.docx]

**Supplementary table 1** The microbiological profile of patients with VAP

| Microbiological profile | Number (%) |
| --- | --- |
| **Gram positive** | **52 (41.3)** |
| *Staphylococcus aureus* | 33 (26.2) |
| *Streptococcus agalatiae* | 1 (0.8) |
| *Streptococcus pneumoniae* | 3 (2.4) |
| *Corynebacterium striatum* | 15 (11.9) |
| **Gram negative** | **71 (56.3)** |
| *Pseudomonas aeruginosa* | 25 (19.8) |
| *Klebsiella pneumoniae* | 17 (13.5) |
| *Klebsiella* spp | 11 (8.7) |
| *Escherichia coli* | 9 (7.1) |
| *Hafnia alvei* | 3 (2.4) |
| *Serratia marcescens* | 2 (1.6) |
| *Haemophilus influenzae* | 2 (1.6) |
| *Acinetobacter baumannii* | 2 (1.6) |
| **Fungi (molds)** | **3 (2.4)** |
| *Aspergillus* spp | 3 (2.4) |
| **Viruses** | **17 (13.5)** |
| *Influenza* | 7 (5.5) |
| *Rhinovirus* | 6 (4.8) |
| *Respiratory Syncytial Virus* | 3 (2.4) |
| *Parainfluenza* | 1 (0.8) |
